# Comparison of the Ventral Approach to the Canine Hip Joint Using Gelpi Retractors and an Elastic O-Ring Wound Retractor

**DOI:** 10.3390/ani13223582

**Published:** 2023-11-20

**Authors:** Piotr Trębacz, Jan Frymus, Mateusz Pawlik, Michał Czopowicz, Anna Barteczko, Aleksandra Kurkowska, Krzysztof Zdeb, Marek Galanty

**Affiliations:** 1 Department of Surgery and Anaesthesiology of Small Animals, Institute of Veterinary Medicine, Warsaw University of Life Sciences-SGGW, 02-776 Warsaw, Poland; jan_frymus@sggw.edu.pl (J.F.); marek_galanty@sggw.edu.pl (M.G.); 2 CABIOMEDE Sp. z.o.o., ul. Karola Olszewskiego 21, 25-663 Kielce, Poland; mateusz.pawlik@cabiomede.com (M.P.); anna.barteczko@cabiomede.com (A.B.); aleksandra.kurkowska@cabiomede.com (A.K.); 3 Division of Veterinary Epidemiology and Economics, Institute of Veterinary Medicine, Warsaw University of Life Sciences-SGGW, Nowoursynowska 159 C Street, 02-776 Warsaw, Poland; michal_czopowicz@sggw.edu.pl; 4 Anicura Legwet Klinika Weterynaryjna Legionowo, Wysockiego 31, 05-120 Legionowo, Poland; krzysztof.zdeb@legwet.pl

**Keywords:** ventral hip approach, Gelpi retractor, elastic O-ring wound retractor, dog

## Abstract

**Simple Summary:**

Currently, there is an increasing emphasis on modifying surgical techniques to reduce iatrogenic damage to the patient’s tissues. Therefore, the aim of our study was to compare the surface area of the surgical wound bed after implementing the ventral approach to the hip joint using two orthogonally inserted Gelpi retractors and an O-ring elastic wound retractor (O-WR). Self-retaining metal retractors, such as Gelpi retractors, are potentially dangerous and can damage retracting tissues. Unlike Gelpi retractors, the O-WR is an atraumatic, self-retaining retractor. Such a device can be an alternative to metal retractors and other methods of soft tissue retraction such as stay sutures. This study included adult large breed dog cadavers. For each cadaver, two hip joints were accessed via the ventral approach without pectinectomy. After retraction of the wound with the pair of Gelpi retractors or the O-WR, digital photographs were taken, with a ruler placed next to the wound. The final step of the analysis was to compare the surface area of the surgical wound bed obtained after the use of Gelpi retractors and the O-WR. In this study, the O-WR provides the same surgical wound bed area as the most commonly used Gelpi retractors.

**Abstract:**

This study included 10 fresh adult cadavers of large breed dogs (6 males and 4 females). Their weight ranged from 25 to 45 kg (mean ± SD: 33.9 ± 6.2 kg). The breeds represented were crossbreed dogs (*n* = 5), German shepherds (*n* = 2), Bernese mountain dogs (*n* = 1), American Staffordshire terriers (*n* = 1), and Gordon setters (*n* = 1). Access to the target area and identification of the femoral head and neck was achieved with two Gelpi retractors inserted orthogonally and with the O-WR in all procedures. In each dog, the approach to the hip joint was made on the left and right sides. There was no significant difference in the area of the surgical wound bed between the two sides using either the Gelpi retractors (−0.52 ± 1.87 cm^2^; CI 95%: −1.86, 0.81 cm^2^; *p* = 0.398) or the O-WR (−0.27 ± 2.34 cm^2^; CI 95%: −1.94, 1.41 cm^2^; *p* = 0.729). The area of the surgical wound bed was 6.28 ± 1.72 cm^2^ (2.72–9.70 cm^2^) for the Gelpi retractors and 6.34 ± 1.81 cm^2^ (4.13–10.77 cm^2^) for the O-WR, and the difference between the Gelpi retractors and the O-WR was not significant (−0.06 ± 1.72 cm^2^; CI 95%: −0.86, 0.74 cm^2^; *p* = 0.879)

## 1. Introduction

The word retractor is derived from the Latin ‘re’ (back) and ‘trahere’ (to draw) [1]. There is a large range of both hand-held and self-retaining retractors. The clear advantage of self-retaining retractors is that it obviates the need for a hand-held instrument and gives a stable clear view of the operative field. One of the most widely used self-retaining retractors in small animal surgery is the Gelpi retractor, which consists of two metal blades held open by a ratchet that is manually locked in the desired opening position. The Gelpi is often used in pairs, with two retractors placed opposite or at right angles to each other [2].

**Figure 1:**
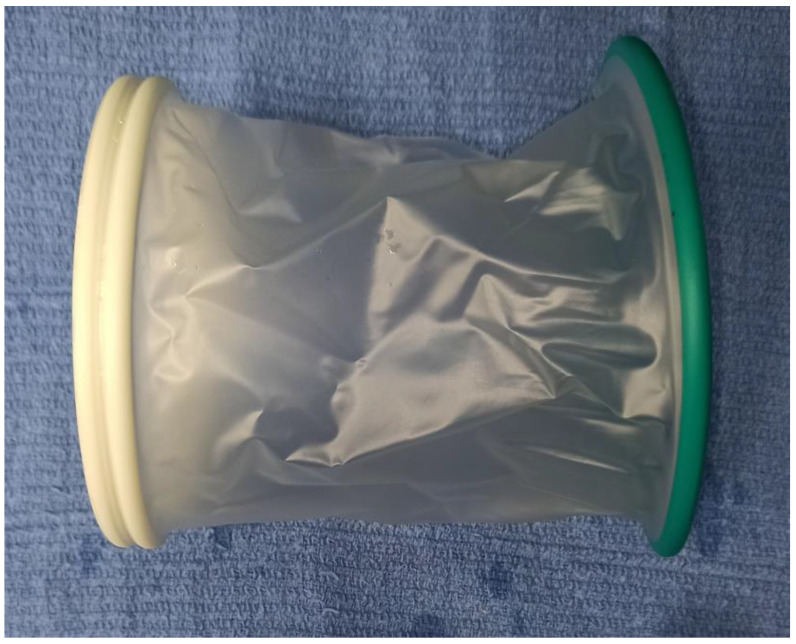
Elastic O-ring wound retractor (O-WR).

An O-ring elastic wound retractor (O-WR) is a single-use, cylinder-shaped device made of two semirigid rings attached by a thin flexible polymer membrane (Figure 1). This device provides atraumatic wound retraction, maximizes the exposure, minimizes the incision size, and thus reduces the risk of wound infection [3,4]. The inner ring of the retractor, placed deep into the wound, is malleable but expands firmly outwards to maintain its circular shape. Its flexibility allows it to be introduced through a small incision and to be placed precisely around the field of dissection. The outer ring is more rigid and while it remains outside the wound, it has an extra advantage of protecting shielded tissue. Once the inner ring is in position, the plastic sheet is drawn through the outer hoop and folded backwards over its circumference. This draws the walls of the surgical wound outwards and creates an opening with stable and wide exposure.

A wide array of soft tissue and orthopaedic surgical procedures can benefit from the use of the O-ring elastic wound retractor, including open and minimally invasive procedures such as lung resection via video-assisted mini-thoracotomy in humans [5], laparoscopic ovariectomy in pets and wildlife [6], and orthopaedic procedures including hip replacement in humans [7].

The ventral approach to the hip joint is useful for open reduction and fixation of femoral head fractures [8], ventro-caudal hip luxation [9], or femoral head and neck ostectomy (FHNO) in small animals. In the case of FHNO, the main advantage of this approach over the more popular craniolateral approach is the preservation of the craniodorsal musculature and soft tissue support structures, which have to take over the function of the hip joint after FHNO [10]. A skin incision is usually made along the pectineus muscle, starting approximately above the coxofemoral joint and continuing to the proximal part of the femur. Sex-related differences in the animal undergoing surgery and the presence of external genitalia have no effect on the surgical technique. To our knowledge, the O-WR has not been used in small animal orthopaedic surgery. Therefore, the aim of this study was to compare the surface area of the surgical wound bed after ventral access to the hip joint using two orthogonally inserted Gelpi retractors and the O-WR. We assumed that there would be no difference in the surface area of the surgical wound bed after insertion of a pair of Gelpi retractors or O-WR.

## 2. Materials and Methods

**Figure 2:**
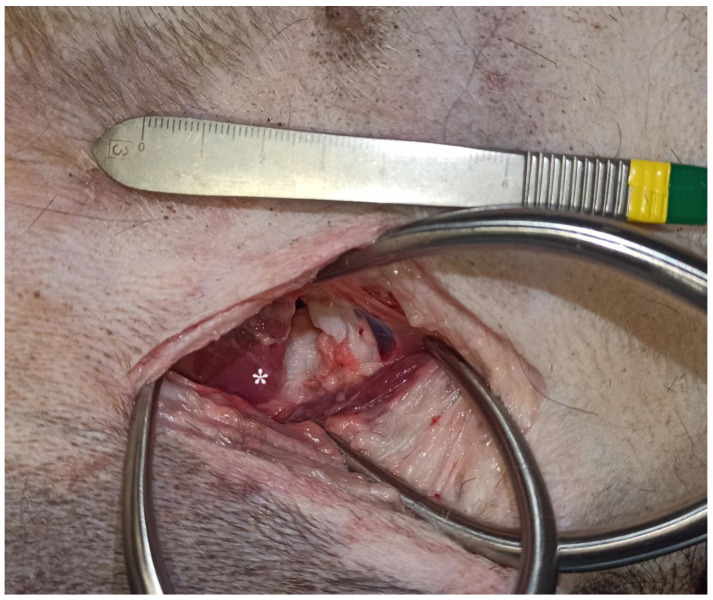
Bernese mountain dog, male. Ventral approach to the right hip joint. The surgical wound was retracted with a pair of Gelpi retractors (head on the top). * Iliopsoas muscle.

**Figure 3:**
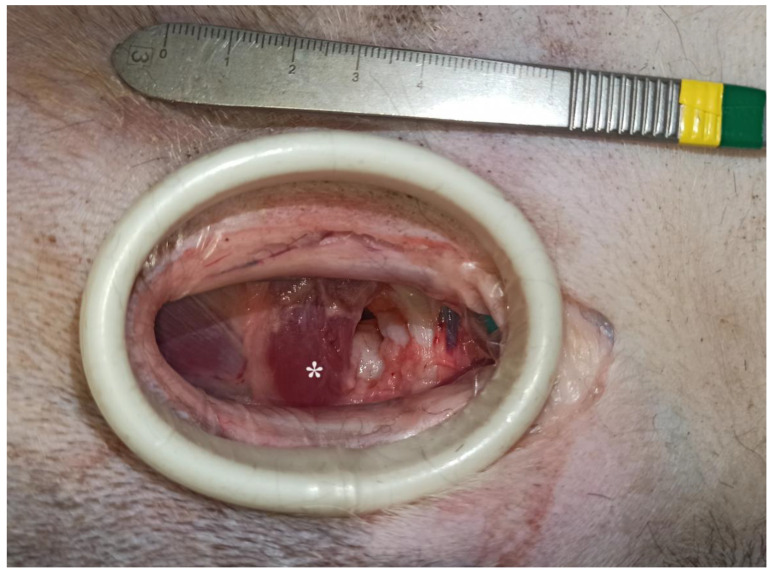
Bernese mountain dog, male. Ventral approach to the right hip joint. The surgical wound was retracted with an elastic O-ring wound retractor (head on the top). * Iliopsoas muscle.

This study included 10 fresh adult cadavers of large breed dogs (6 males and 4 females). Their weight ranged from 25 to 45 kg (mean ± SD: 33.9 ± 6.2 kg). All dogs had died or were euthanized for reasons unrelated to this study. The breeds represented were crossbreed dogs (*n* = 5), German shepherds (*n* = 2), Bernese mountain dogs (*n* = 1), American Staffordshire terriers (*n* = 1), and Gordon setters (*n* = 1). For each cadaver, two hip joints were accessed via the ventral approach. No pectinectomy was performed. Each surgical wound was observed on the same cadaver to evaluate the two methods of wound retraction. Gelpi retractors and O-WR were assessed separately on each wound. This was carried out to eliminate variability in soft tissue tension, elasticity, and surgical wound length between cadavers. These differences can affect comparisons of the degree of surgical wound distraction. With the dog in dorsal recumbency, the skin incision was made parallel to the pectineus muscle, starting above the hip joint and continuing for approximately 1/3 of the length of the thigh. After releasing the fascia to the level of the femoral artery and vein branches, a blunt dissection was continued between the pectineus muscle and the adductor longus muscle. Deep wound retraction was then performed using two orthogonally placed Gelpi retractors: 130 mm long and 60 mm wide (Rudolf, Fridingen Germany). After exposure of the lesser trochanter and the iliopsoas muscle, the joint capsule was sharply incised to expose the femoral head and neck. Then, the retractors were repositioned to achieve optimal exposure, and a ruler was placed at the margin of the wound. Digital photographs with a smartphone (Xiaomi 12 Lite, Beijing China) were taken from the surgeon’s perspective. These images were then imported into a computer program, GIMP 2.10.34 (www.gimp.org), and the exposed surface area of the surgical wound bed was calculated. The area of the wound bed was outlined manually and coloured blue. The next step was to calibrate the images. Based on the 1 cm section of the ruler present in each photo, the number of corresponding pixels was measured. The photos were scaled so that 1 cm corresponded to 100 pixels, which in practice meant that each pixel represented a square with a side of 0.1 mm (area of 0.01 mm^2^). Then, the number of visible blue pixels was counted and converted to surface area in cm^2^ (1 cm^2^ = 10,000 pixels). After removal of the Gelpis, the O-WR 60 × 70 × 150 mm (RingProtect, Grena, Brentford, UK) was inserted. No further incisions were made to insert the elastic retractor. Tension was applied to the sleeve by bringing the outer ring up to the inner ring. After retraction of the wound with the O-WR, digital photographs with a ruler placed next to the wound were taken (Figure 2 and Figure 3). The final step of the analysis was to compare the surface area of the surgical wound bed obtained after the use of two Gelpi retractors and the O-WR.

### Statistical Methods

Numerical data as well as the differences between paired data (left vs. right side and the Gelpi retractors vs. the O-WR) were examined for normality using the normal probability Q–Q plots and the Shapiro–Wilk W test. As the assumption of distribution normality was satisfied, numerical data were summarized using the arithmetic mean, standard deviation (±SD), and range, and the surgical wound area was compared between sides and between retractors using the paired *t*-test and presented as the mean (±SD) difference with a 95% confidence interval (CI 95%). The required number of dogs was calculated to ensure at least 80% power of detecting the mean difference between retractors of at least 2 cm^2^, assuming a SD of the difference equal to 2 cm^2^. The alternative hypothesis was two-sided, and the significance level (α) was set at 0.05. Statistical analysis was performed in TIBCO Statistica 13.3 (TIBCO Software Inc., Palo Alto, CA, USA).

## 3. Results

**Figure 4:**
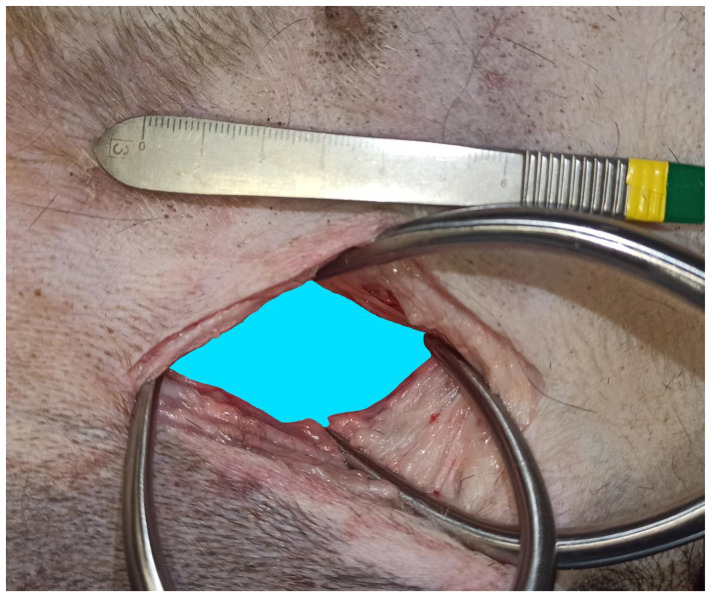
Bernese mountain dog, male. The area of the wound bed retracted using a pair of Gelpi retractors was outlined manually and coloured blue. The number of visible blue pixels was counted and converted to surface area in cm^2^ (57,842 blue pixels = 5.78 cm^2^).

**Figure 5:**
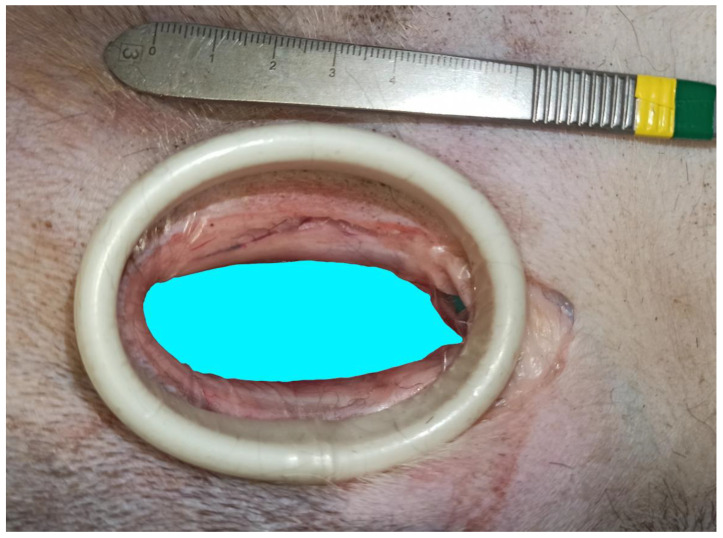
Bernese mountain dog, male. The area of the wound bed retracted using an elastic O-ring retractor was outlined manually and coloured blue. The number of visible blue pixels was counted and converted to surface area in cm^2^ (79,788 blue pixels = 7.98 cm^2^).

Access to the target area and identification of the femoral head and neck was achieved with two Gelpi retractors inserted orthogonally and with the O-WR in all procedures. In each dog, the approach to the hip joint was made on the left and right sides. There was no significant difference in the area of the surgical wound bed between the two sides using either the Gelpi retractors (−0.52 ± 1.87 cm^2^; CI 95%: −1.86, 0.81 cm^2^; *p* = 0.398) or the O-WR (−0.27 ± 2.34 cm^2^; CI 95%: −1.94, 1.41 cm^2^; *p* = 0.729) (Table 1). The area of the surgical wound bed was 6.28 ± 1.72 cm^2^ (2.72–9.70 cm^2^) for the Gelpi retractors and 6.34 ± 1.81 cm^2^ (4.13–10.77 cm^2^) for the O-WR, and the difference between the Gelpi retractors and the O-WR was not significant (−0.06 ± 1.72 cm^2^; CI 95%: -0.86, 0.74 cm^2^; *p* = 0.879) (Figure 4 and Figure 5), (Table 1).

## 4. Discussion

In our study, both types of retractors allowed ventral access to the hip joint in large breed dogs. There was no significant difference in the area of the surgical wound bed between the Gelpi retractors and the O-WR, confirming our hypothesis. Metal self-retaining retractors are potentially hazardous and can damage distracted tissues. The injury caused by retractors mostly appears to result from compression, which increases interstitial fluid hydrostatic pressure, leading to interstitial oedema and diminished blood flow in compressed tissue [11]. In addition to tissue compression, the pointed tips of Gelpi blades can be dangerous to vital structures. These retractors are versatile surgical instruments widely used in veterinary surgery, including orthopaedic surgery on dogs, cats, sheep [12], and other species. These retractors have sharp tips and care must be taken when inserting and removing them. Pang et al. [13] described the case of a girl with scoliosis who developed a massive haemothorax as a result of injury to the intercostal vasculature caused by the Gelpi retractor used during surgery. The authors of this study have had a similar experience of vascular injury. During the ventral approach to the hip joint, the blades of the Gelpi retractor can damage the medial circumflex vasculature.

In contrast to the Gelpi retractor, the O-WR is described as an atraumatic self-retaining retractor [14]. Such a device can be an alternative to metal retractors and other methods of soft tissue distraction, such as stay sutures [15]. The expandable circular structure of the O-WR distributes the traction force evenly around the soft tissues, providing atraumatic retraction and wound protection over 360° of the wound margins. Circumferential elastic retraction maximizes the working area for improved visualization and exposure. Thin and elastic elements of the retractor lying inside the wound do not interfere with any surgical instruments that may be used [15]. The O-WR has been shown to ensure good exposure of tissues and high visibility of the surgical area in thyroid and parathyroid surgery [16], oral and maxillofacial surgery [17,18], and obstetrics surgery [19]. In addition, the O-WR is an inexpensive disposable device produced by a number of manufacturers. The cost of each elastic retractor used in our study was approximately EUR 8. This marginally increases the total cost of surgery and, in our opinion, should not be taken into account when choosing between reusable Gelpi retractors and the O-WR. Since our study was carried out on cadavers, we could not assess the risk of surgical site infection (SSI), safety, intraoperative bleeding, surgical times, and postoperative pain, which have been described as benefits of using the O-WR in human medicine. For example, Lois-Ortega et al. [16] found that the homogeneous tension created by the O-WR favoured skin haemostasis. Similar results can be expected when performing surgery on live animals. One of the most important methods of preventing SSI is mechanical wound edge protection. O-WR provides a physical barrier between the wound edge and the rest of the surgical field. Proponents of the ring retractor claim increased wound edge moisturisation, less bruising, and reduced local trauma compared to standard metal retractors alone. [19,20]. It is difficult to assess the preventable fraction in veterinary medicine, but it is reasonable to assume that a substantial percentage of SSIs in veterinary surgery are potentially preventable [21] and that the O-WR might play an important role in this process. Recent studies on the effectiveness of the O-WR in preventing SSI in human medicine have shown that it is more effective than traditional metal retractors in colorectal surgery [22,23,24] and equally functional (or effective) as traditional metal retractors in caesarean sections [25,26]. One such study [25] revealed as high as an eight-fold reduction in the risk of SSI with the O-WR compared to traditional metal retractors. Its use has also been linked to less postsurgical pain [27] and a higher quality of life for patients [28]. A prospective randomized controlled trial is warranted to evaluate these outcomes and to confirm the potential use of the ring retractor for future orthopaedic procedures in small animals.

Our study has one major limitation: the Gelpi retractors were used first. The Gelpis may have stretched the tissue and therefore increased the exposure for the O-WR. As we did not find statistically significant differences between the area of the surgical wound bed after the use of a pair of Gelpi retractors and the O-WR, we considered this to be insignificant. However, it is conceivable that the stretching of the Gelpi retractors first increased the wound surface area, and it is possible that if the sequence were reversed, the exposed area after use of the O-WR would be smaller than that of the Gelpi retractors.

## 5. Conclusions

In canine hip surgery, the O-WR provides the same area of surgical wound bed as the most commonly used Gelpi retractors. As the O-WR is known to be significantly less traumatic, we recommend its routine use in the ventral approach to the hip joint in large breed dogs.

## Figures and Tables

**Table 1:** Characteristics of the cadavers and the surface area of the surgical wound bed.

Cadaver Characteristics	Side	Area in cm^2^ (Pixels) ^a^ Obtained Using Retractors:	
		Gelpi	O-Ring (O-WR)
Crossbreed, female, 28 kg	Left	2.72 (27,166)	4.84 (48,400)
	Right	3.09 (30,877)	4.18 (41,787)
Crossbreed, female, 30 kg	Left	4.35 (43,501)	7.13 (71,332)
	Right	6.52 (65,188)	5.97 (59,689)
Bernese mountain dog, male, 37 kg	Left	6.12 (61,199)	6.46 (64,583)
	Right	5.78 (57,824)	7.98 (79,788)
German Shepherd, male, 38 kg	Left	6.50 (64,999)	5.29 (52,936)
	Right	7.19 (71,927)	7.08 (70,800)
American Staffordshire terrier, male, 29 kg	Left	6.56 (65,609)	6.51 (65,120)
	Right	5.34 (53,410)	5.28 (52,752)
Crossbreed, female, 35 kg	Left	6.97 (69,705)	5.20 (52,028)
	Right	7.94 (79,373)	5.99 (59,934)
German Shepherd, female, 32 kg	Left	6.16 (61,604)	4.43 (44,301)
	Right	6.54 (65,420)	4.13 (41,319)
Crossbreed, male, 40 kg	Left	5.70 (56,992)	6.64 (66,445)
	Right	9.70 (97,013)	10.77 (107,711)
Gordon setter, male, 45 kg	Left	7.54 (75,394)	5.14 (51,428)
	Right	8.71 (87,076)	7.27 (72,666)
Crossbreed, male, 25 kg	Left	7.59 (75,908)	10.46 (104,584)
	Right	4.64 (46,354)	6.10 (60,978)
Descriptive statistics			
Arithmetic mean ± standard deviation (range)	Left	6.02 ± 1.49 (2.72–7.6)	6.21 ± 1.74 (4.43–10.46)
	Right	6.54 ± 1.96 (3.09–9.70)	6.48 ± 1.96 (4.13–10.77)
	Overall	6.28 ± 1.72 (2.72–9.70)	6.34 ± 1.81 (4.13–10.77)

^a^ 1 cm^2^ = 10,000 pixels.

## Data Availability

The data presented in this study are available on request from the corresponding author.
